# Characteristics of Mobile Health Interventions for Repetitive Negative Thinking: Protocol for a Scoping Review

**DOI:** 10.2196/72138

**Published:** 2026-01-23

**Authors:** Judith Martens, Thomas Forkmann, Inken Höller

**Affiliations:** 1 Department of Clinical Psychology and Psychotherapy University of Duisburg-Essen Essen Germany; 2 Department of Clinical Psychology and Psychotherapy Charlotte Fresenius Hochschule Düsseldorf Germany

**Keywords:** mobile health, mHealth, digital health, repetitive negative thinking, digital interventions, transdiagnostic, rumination, scoping review

## Abstract

**Background:**

Many people are affected by mental disorders. A transdiagnostic symptom and risk factor for most mental disorders is repetitive negative thinking (RNT). Psychotherapy can reduce RNT, but most people in need either do not receive psychotherapy or face long waiting times. In addition, people at risk for developing mental disorders do not receive psychotherapy. Mobile health (mHealth) interventions could overcome treatment barriers and support people at risk.

**Objective:**

This scoping review aims to identify existing mHealth interventions for RNT and to give an overview of their characteristics regarding content, context, and technical features. Another aim is to identify which outcomes and questionnaires are used to measure RNT.

**Methods:**

The scoping review will be conducted according to the JBI (Joanna Briggs Institute) methodology for scoping reviews and will be reported in accordance with the PRISMA-ScR (Preferred Reporting Items for Systematic Reviews and Meta-Analyses Extension for Scoping Reviews) guidelines. English-language, peer-reviewed literature involving mHealth interventions aimed at reducing RNT from 2003 onward will be included. A comprehensive search will be conducted in the following databases: PsycInfo, PubMed, Scopus, and Web of Science. Two independent reviewers will conduct a 2-stage blinded screening process (screening of title or abstract and full text) of the articles according to the inclusion criteria. A data extraction table will be used to extract information on the technology, content, delivery, accessibility, usability, feedback, and outcome measures of the interventions. Data charting, including coding and grouping, will follow an iterative process. The charted data will then be synthesized descriptively.

**Results:**

Data collection began in February 2025 and is now complete, with 21 included articles meeting the inclusion criteria. As of December 2025, data charting is currently underway, and data synthesis will begin shortly. The final scoping review is expected to be submitted to a peer-reviewed journal by April 2026.

**Conclusions:**

The scoping review should lead to a better understanding of the conceptual possibilities and commonalities of current mHealth interventions for the transdiagnostic symptom RNT. It should provide starting points for systematic reviews and for the development of transdiagnostic mHealth interventions.

**International Registered Report Identifier (IRRID):**

PRR1-10.2196/72138

## Introduction

### Epidemiology

Epidemiological studies show that 1 in 3 persons is affected by a mental disorder in their lifetime, and 1 in 5 persons has experienced a mental disorder within the last 12 months [1,2]. Therefore, the prevalence of mental disorders in the population is alarmingly high, and the burden, societal impacts, and costs are massive [1]. Moreover, mental disorders do not only impair quality of life [3] but also serve as a risk factor for suicide [4] and are related to a greater risk of developing chronic physical illnesses [5]. The detection and especially the treatment of symptoms are therefore indispensable. In particular, symptoms that occur across different disorders should be targeted for treatment.

### Repetitive Negative Thinking

A transdiagnostic symptom that occurs in almost all mental disorders is repetitive negative thinking (RNT) [6]. Ehring and Watkins [6] define RNT “as repetitive thinking about one or more negative topics that is experienced as difficult to control.” Two common forms of RNT are rumination and worry. Both have so far mostly been recorded in the literature on a disorder-specific basis [6]. For example, rumination was usually measured in the context of depression [7], and worry was usually measured in the context of generalized anxiety disorder [8]. However, as RNT has more similarities than differences between different disorders, it is argued that RNT can be referred to as a transdiagnostic process [6]. Ehring and Watkins [6] found evidence that RNT occurs in up to 13 different disorders, for example, depression, social phobia, and obsessive-compulsive disorder.

Furthermore, RNT can be found not only as a transdiagnostic symptom but also as a risk factor for the development of a mental disorder, which may also be causal for the maintenance of mental disorders. Studies have shown that rumination serves as a transdiagnostic risk factor impacting anxiety, depression, psychosis, insomnia, and impulsive behaviors [9]. In addition, rumination intensifies psychopathological conditions by amplifying and protracting negative affective states, impeding effective problem-solving and instrumental behaviors, and diminishing responsiveness to evolving circumstances [9]. These findings demonstrate the high relevance of RNT as a treatment target in psychotherapy already at an early stage and not only when the mental disorder has already manifested itself.

There are several treatment approaches in psychotherapy offering different interventions that can reduce RNT, for example, cognitive behavioral therapy (CBT) [10]. However, only individuals who are actively engaged in psychotherapy can benefit from these approaches. Studies show that most people with mental disorders do not receive treatment [11-13] or drop out of treatment [14]. Furthermore, the waiting time in Germany for those who eventually receive psychotherapy is, on average, 4.6 months [15]. In addition, individuals, who engage in RNT and may be at risk for developing a mental disorder, may not inherently perceive the imperative for comprehensive therapeutic involvement. This is in line with a study by Andrade et al [16] who found that the greatest barriers to receiving and staying in treatment are low perceived need and attitudinal barriers. For those who recognized a need, one of the most common reasons for not getting treatment was the wish to deal with the problem themselves. Self-stigmatization and label avoidance could also be linked to this desire [16]. Another reason for the lack of treatment are structural barriers, such as finance and availability (particularly in developing countries), especially for severe disorders [16].

### Mobile Health

Therefore, the reduction of barriers for receiving fast and low-threshold treatment that addresses RNT is of high importance. One option could be to look for new interventions beyond the classic face-to-face treatments. One approach that has gained prominence in overcoming barriers, particularly in the context of the COVID-19 pandemic, is the use of mobile health (mHealth) interventions [17], first introduced in 2003 [18,19]. mHealth uses mobile and wireless technologies for health, such as smartphones and tablets [20]. These interventions can vary in structure, components, content, duration, and other characteristics. One specific type of mHealth intervention is ecological momentary interventions (EMIs) [21]. EMIs use real-time data from ecological momentary assessment [22]. Through real-time monitoring and data collection in individuals’ environments [22], EMIs enable the delivery of treatments within these settings, offering support when needed [21].

An RNT is not only a symptom but also a risk factor for the development of mental disorders [7], and low-threshold treatment is needed for people at risk. Therefore, EMI can offer an important opportunity for the treatment of RNT and the prevention of mental disorders. EMIs provide support when needed, which is critical because RNT fluctuates over the course of time [23]; it has been shown to occur predominantly in the evening in individuals with severe depression [23]. As a treatment option for people with mental disorders, EMI could additionally help to implement techniques learned in psychotherapy in everyday life, such as supporting the practice of techniques as homework, which can be difficult in psychotherapy for various reasons, including patients’ characteristics such as low motivation or self-doubts [24]. mHealth interventions can overcome this problem by being accessible anytime and anywhere. This could be beneficial not only for people who are receiving treatment but also for those who are waiting for treatment or have already finished treatment in terms of relapse prevention.

mHealth interventions have gained attention in recent years. Previous reviews about mHealth interventions in the context of mental health have focused on the use and effects of smartphone interventions on mental health in general [25], on specific disorders such as depression [26] or anxiety disorders [27], as well as on the management of suicidal thoughts and behavior [28]. There are also reviews on the use and efficacy of certain interventions for RNT, such as digital mindfulness-based interventions [29].

However, there is a gap in the overview of available mHealth interventions for RNT. As there is a wide range of variations in mHealth interventions (in terms of duration, type of intervention, frequency, content, components, intensity, etc), it seems useful to first use a broader scoping review to provide an overview of mHealth interventions that address RNT and their specific characteristics. Gaps and potential for improvement of mHealth interventions will be identified and discussed to establish a foundation for the development of subsequent interventions.

### Review Questions

The review questions are as follows:

1. What mHealth interventions for RNT are currently available for people who experience RNT?
2. What are the contents, contexts, and technical features of these interventions?
3. What outcomes and questionnaires have been used to measure RNT within the mHealth interventions and as an outcome variable to evaluate the efficacy of the interventions in relation to RNT?

## Methods

### Overview

To ensure quality and transparency of the scoping review, this scoping review protocol and the final scoping review will be conducted in accordance with the JBI (Joanna Briggs Institute) methodology for scoping reviews [30] and scoping review protocols [31]. The final review will also be reported in accordance with the PRISMA-ScR (Preferred Reporting Items for Systematic Reviews and Meta-Analyses Extension for Scoping Reviews) guidelines [32]. The completed PRISMA-ScR checklist can be found in Multimedia Appendix 1. The mHealth evidence reporting and assessment guidelines [20] will be used as the basis for the evaluation criteria for the interventions. The scoping review is preregistered at OSF [33]. Pilot testing of inclusion criteria was conducted after registration but before screening on a small sample of studies to ensure clearly defined boundaries. The inclusion criteria were then further specified before the screening process started. The refined criteria are reported in the Eligibility Criteria section.

### Ethical Considerations

As no human participants were involved in this research, no ethics approval is required.

### Eligibility Criteria

Eligibility criteria are based on the population-concept-context framework, as recommended for systematic scoping reviews [31] (Table 1).

**Table 1:** Population-concept-context (PCC) framework.

PCC framework	Definition
Population	People experiencing RNT^a^
Concept	mHealth^b^ interventions that aim to reduce RNT
Context	User’s everyday life and real-world environment

^a^RNT: repetitive negative thinking.

^b^mHealth: mobile health.

#### Population

The target population of the mHealth interventions must consist of people who experience RNT. The intervention study must explicitly include participants who exhibit RNT, as verified through appropriate means. This must include the use of specific RNT questionnaires (eg, Perseverative Thinking Questionnaire [34]) or the use of diagnostic or symptom-based questionnaires for disorders in which RNT is a core feature—this includes depression and generalized anxiety disorder [6]. Apart from the presence of RNT, no further restrictions were applied regarding sample type—which means that all kinds of sample types, for example, clinical and nonclinical populations, were included, irrespective of specific diagnoses, as RNT is conceptualized as transdiagnostic [6]. In terms of age, we expanded the inclusion criteria from including all mHealth interventions for RNT for participants above the age of 18 years, as stated in the scoping review protocol, to adolescents. This is because research indicates that RNT already develops in adolescence [35]. Therefore, providing interventions early is essential for the prevention of long-term psychological difficulties. The inclusion age of participants was determined based on other research on RNT in adolescence [35], and therefore, people aged 10 years and older will be included.

#### Concept

The concept of interest was mHealth interventions that aim to reduce RNT. mHealth was defined such that the interventions had to be delivered via smartphones or tablets or at least had to be accessible with smartphones or tablets, even if they were web based. Computer- or web-based interventions that were only accessible via computers or laptops were not considered. Articles had to aim to address RNT. To clarify this inclusion criterion, the following restrictions were set before the search process started: the aim of the intervention had to be stated as reducing some form of RNT (eg, RNT, rumination, and worry). The intervention had to have been developed to change RNT or at least a theoretical rationale or theoretical model had to be provided as to why this intervention was intended to reduce RNT. Articles or interventions were excluded if the intervention was not explicitly designed for RNT, and there was no theoretical and scientific justification as to why the intervention was intended to help reduce rumination. Articles were also not included if they contained only assessments.

Another concept of interest was the outcomes and assessment tools to assess RNT within these interventions. Therefore, studies had to include an outcome measure that assesses RNT, either as a primary or secondary outcome. Any form of quantitative assessment was eligible regardless of whether the measurement tool was validated. The study had to report on RNT as part of the intervention outcomes.

#### Context

There were no restrictions in terms of geographic context or setting. As the idea was to receive the intervention anywhere and anytime, the intervention had to be available to the participants at all times.

#### Types of Evidence Sources

As the scoping review will focus on intervention design without assessing efficacy, all study designs were included. Only peer-reviewed, full-text articles published in or translated into English were considered. When an intervention appeared in both a review and its primary source, the primary source was prioritized to avoid duplication. Gray literature and other nonpeer-reviewed sources were not included.

### Search Strategy

For this review, a search was conducted in the following electronic library databases: PsycInfo, PubMed, Scopus, and Web of Science. The concepts of interests were mHealth interventions and RNT. The search strategy was based on previous literature on the concepts of interest [6,36-38]. As research has shown that worry and depressive rumination belong to a common construct [6], both terms were included. When searching for sources of evidence, articles published between 2003 and shortly before the scoping review was constructed were considered because 2003 is the year when the first paper was published that defined and introduced the term mHealth [18,19]. The full search string for each database can be found in Multimedia Appendix 2.

The reference lists of relevant articles were also searched, and relevant articles were included. If new relevant terms appeared during the search, they were added to the search string and will be reported in the review.

### Study Selection

First, all articles identified through the search were downloaded and imported into the reference manager Zotero [39]. After that, all identified articles were uploaded to the online tool Rayyan [40]. Next, duplicates will be removed. The remaining articles will then be screened to determine if they meet the inclusion criteria. First, the titles and abstracts were screened by 2 blinded reviewers according to the inclusion criteria. The blinding was then lifted, and conflicts and uncertainties were discussed, and more reviewers were consulted if no consensus could be reached. Full texts were uploaded to Rayyan, the reviewers were then reblinded, and the full texts of the remaining articles were reviewed according to the inclusion criteria. Conflicts and uncertainties were discussed again, and if no agreement could be reached, more reviewers were again consulted until consensus was reached. The reasons for exclusions will be documented and reported in the review. References for the final selection of articles were uploaded to Zotero [39] and stored in a dedicated folder. To represent the inclusion and exclusion process, the selection procedure will be mapped and illustrated using a PRISMA (Preferred Reporting Items for Systematic Reviews and Meta-Analyses) flow diagram [41].

### Data Extraction

First, the data to be extracted are basic study information (eg, author, title, publication year, and location) and sample characteristics (eg, age, gender, and target group). Following the mHealth evidence reporting and assessment guidelines [20], a data extraction table has been designed detailing the data to first evaluate the interventions in case of content, context, technical features, and second, to map which outcomes and assessment tools are used to measure RNT (Table 2). One subsection, called “tailoring,” indicates whether the intervention is an EMI, that is, whether real-time data are used to adapt the interventions. The described table draft will be tested on a subset of studies to evaluate and adjust or expand if necessary. The final table will be documented in the final scoping review. The extracted data from the relevant articles were added verbatim to the data extraction table. The extraction process is being conducted by 1 reviewer (JM), with support from another reviewer (research assistant). In case of disagreements, a third reviewer (IH) is being consulted.

**Table 2:** Data extraction table for intervention evaluation criteria.

Domain and evaluation criteria			Description with example
**Technology platform**			
	Program used	This item describes the used program, for example, a specific app.	
	Requirements	This item describes the technical requirements for the program, for example, the operating system such as iOS or Android.	
**Content**			
	Therapeutic approach or intervention strategies	This item describes the therapeutic approach of the intervention, for example, cognitive behavioral therapy.	
	Components	This describes single components of the intervention, for example, psychoeducation, self-monitoring.	
	Communication	This evaluates how communication with the participant takes place, for example, whether there is human contact or with an artificial intelligence.	
**Delivery**			
	Tailoring	This records whether real-time data are collected and if so, how they are used to customize the intervention, for example, by offering certain interventions.	
	Mode and design of components	This describes the mode and design of the components, that is, the way in which the components are presented, for example, with text messages, videos, or audio.	
	Timing of components	This describes at what time the components take place, for example, randomly or in the morning.	
	Frequency of components	This describes how often the components take place, for example, 5 times a day or every hour.	
	Duration of components	This describes how long the single components take, for example, 10 min for psychoeducation.	
	Duration of intervention	This describes how long the whole intervention takes, for example, 7 d.	
**Accessibility**			
	Costs	This describes whether the intervention costs anything and, if so, how much.	
	Barriers	This describes whether there are barriers that restrict the use of the intervention, for example, an internet connection.	
	Language	This describes in which languages the intervention is available or whether it is possibly language free.	
**Usability**			
	User involvement in development	This describes whether and how the users were involved in the development of the intervention, for example, through focus groups.	
	Adherence	This describes the degree to which participants follow and complete the intervention.	
**Feedback**			
	User satisfaction	This describes user satisfaction with the intervention, for example, recorded via questionnaires.	
**Outcomes**			
	Construct	This describes the construct of interest that is to be reduced, for example, depressive rumination.	
	Operationalization	This describes the assessment tool that is intended to capture the construct of interest, for example, Ruminative Response Scale.	

### Data Charting and Synthesis

After data were added verbatim to the data extraction table according to the predefined categories (technology, content, delivery, accessibility, usability, feedback, and outcomes), the data will be charted by coding and grouping using an iterative process. New relevant categories that emerged during analysis will be added, existing categories will be refined, expanded, or merged, and missing information will be marked. Terms and concepts will be standardized, for example, CBT for cognitive behavioral therapy or manual for cognitive behavioral therapy.

The charted data will be synthesized using a descriptive approach with regard to the research questions and presented using text and tables. Highlights and key findings, such as similarities, differences, or special features regarding the interventions, will be reported.

## Results

The study selection process is completed, and 21 articles meeting the inclusion criteria have been identified. The complete inclusion and exclusion process will be illustrated in the final scoping review using a PRISMA flow diagram [41]. Data extraction has been completed, and data charting is underway, with data synthesis starting soon. Data synthesis is expected to be completed by February 2026, and the manuscript is expected to be finalized by April 2026. It will be submitted to an international peer-reviewed journal.

## Discussion

The aim of this scoping review is to provide an overview of existing mHealth interventions that address RNT and their specific characteristics. Hypothesized main findings include heterogeneity in the construction of the identified mHealth interventions of RNT. As data extraction and synthesis are still pending, no final results can yet be presented. However, we expect differences in the (1) technology, (2) content, (3) delivery, (4) accessibility, (5) usability, (6) feedback, and (7) outcome measures of the interventions.

### Anticipated Findings

Regarding technology, there exist multiple different platforms, which are used to implement mHealth interventions, such as mPath [42] or movisensXS [43]. The content of the interventions might differ due to numerous available approaches for RNT treatment, such as CBT or mindfulness [44]. The delivery of interventions will likely vary, particularly in terms of length and number of delivery as common suggestions in mHealth study designs are lacking, for example, within mHealth for youth [45]. The accessibility might be restricted, especially for older adults [46] and populations in low- and middle-income countries [47]. In terms of usability, it is assumed that adherence might be insufficient [48] and information on adherence and user engagement with the interventions might be lacking [49]. Regarding feedback, assessments of user satisfaction are expected to be heterogeneous, and measures might not have been validated [50]. Finally, outcome measures are expected to vary across studies, reflecting the diversity of instruments available, such as the Perseverative Thinking Questionnaire [34], the Ruminative Response Scale [51], or the Penn State Worry Questionnaire [52].

Another presumed finding is that reporting of information on different aspects of the implemented interventions may be insufficient in many studies. Prior research has, for example, found that safety aspects are insufficiently reported [53] and that information on user engagement is often underreported [49]. Research has also shown that information on the decision-making basis for the design and choice of interventions is lacking and that future studies should empirically justify the design and selection of intervention components [36].

If the results of the final scoping review are as expected, it will be crucial for future research to develop comprehensive study protocols; provide all relevant information; and use existing guidelines and checklists to ensure comprehensibility, transparency, and reproducibility.

### Limitations

Although this research is the first to provide an overview of existing mHealth interventions for RNT and thus offers important starting points for further research, some limitations must be considered. As this paper is a scoping review, the efficacy of included interventions is not evaluated and no conclusions of effectiveness of the individual components or contents can be drawn. Given the wide range of design possibilities, it was the goal to first provide an overview of the designs and implementations of mHealth interventions for RNT before evaluating them.

Evaluation should be relevant for future research steps aiming at further advancing the prevention of mental health disorders by treating the risk factor RNT. Future studies might also need to differentiate more closely between interventions for RNT with regard to subgroups of interventions (eg, CBT interventions vs mindfulness).

### Conclusions

All in all, mHealth interventions are promising to address gaps in care, provide supplementary support, and enhance access to mental health resources for individuals at risk or individuals who might otherwise face barriers to psychotherapy. The results of the final scoping review will be used as a starting point for new research within our academic institution to develop transdiagnostic mHealth interventions and later investigate their usability and effectiveness.

## Appendix

Multimedia Appendix 1 PRISMA-ScR checklist.

Multimedia Appendix 2 Search strategy.

## Abbreviations

CBT: cognitive behavioral therapy
EMI: ecological momentary intervention
GAIDeT: Generative AI Delegation Taxonomy
JBI: Joanna Briggs Institute
mHealth: mobile health
PRISMA: Preferred Reporting Items for Systematic reviews and Meta-Analyses
PRISMA-ScR: Preferred Reporting Item for Systematic Reviews and Meta-Analyses for Scoping Reviews
RNT: repetitive negative thinking

## Data Availability

All data used in the final scoping review consist of previously published studies, which will be cited in the manuscript. The data extraction table summarizing study characteristics and key findings will be available from the corresponding author on reasonable request.
