# Supplementary material for: Characteristics of Mobile Health Interventions for Repetitive Negative Thinking: Protocol for a Scoping Review
Source: JMIR Res Protoc. 2026 Jan 23;15:e72138. doi: 10.2196/72138 (PMC12881901; doi:10.2196/72138)
Supplement: Multimedia Appendix 2 [file resprot_v15i1e72138_app2.docx]

| **Database** | **Search Strategy** | **Filters** |
| --- | --- | --- |
| PsychInfo through Ovid | (("interven*" or "training" or "treat*" or "therap*" or "reduc*" or "enhanc*" or "program" or "support" or "improv*" or "target*") and ("mobile health" or "mhealth" or "mobile application" or "app" or "smartphone" or "tablet" or "ecological momentary" or "just in time" or "EMI" or "JITAI") and ("repetitive negative thinking" or "RNT" or "ruminat*" or "worry" or "negative thoughts")).mp. [mp=title, abstract, heading word, table of contents, key concepts, original title, tests & measures, mesh word] | Year (2003 to current (06/02/25); without map term to subject headings |
| PubMed | ("interven*" OR "training" OR "treat*" OR "therap*" OR "reduc*" OR "enhanc*" OR "program" OR "support" OR "improv*" OR "target*")  AND  ("mobile health" OR "mhealth" OR "mobile application" OR "app" OR "smartphone" OR "tablet" OR "ecological momentary" OR "just in time" OR "EMI" OR "JITAI")  AND  ("repetitive negative thinking" OR "RNT" OR "ruminat*" OR "worry" OR "negative thoughts") | Year (2003 to current (05/02/25); ALL FIELDS |
| Scopus | ("interven*" OR "training" OR "treat*" OR "therap*" OR "reduc*" OR "enhanc*" OR "program" OR "support" OR "improv*" OR "target*")  AND  ("mobile health" OR "mhealth" OR "mobile application" OR "app" OR "smartphone" OR "tablet" OR "ecological momentary" OR "just in time" OR "EMI" OR "JITAI")  AND  ("repetitive negative thinking" OR "RNT" OR "ruminat*" OR "worry" OR "negative thoughts") | Year (2003 to current (05/02/25); Search within Article title, Abstract, Keywords |
| Web of Science | TS=("interven*" OR "training" OR "treat*" OR "therap*" OR "reduc*" OR "enhanc*" OR "program" OR "support" OR "improv*" OR "target*")  AND  TS=("mobile health" OR "mhealth" OR "mobile application" OR "app" OR "smartphone" OR "tablet" OR "ecological momentary" OR "just in time" OR "EMI" OR "JITAI")  AND  TS=("repetitive negative thinking" OR "RNT" OR "ruminat*" OR "worry" OR "negative thoughts") | Year (2003 to current (05/02/25) |
